# African guidelines for diagnosis and management of polyarticular juvenile idiopathic arthritis: PAFLAR initiative

**DOI:** 10.1186/s12969-025-01076-5

**Published:** 2025-03-15

**Authors:** Mohammed Hassan Abu-Zaid, Angela Nyangore Migowa, Hanna Lishan Kassa, Wassila Messadi, Yassmine Taha, Yaninga Halwani Fuseini, Madeleine Ngandeu, Yasser El Miedany, Michael Hofer, Wafa Hamdi, Temesgen Teferi Libe, Ali Sobh, Waleed Hassan, Yasmine Makhlouf, Ayodele Faleye, Soad Hashed, Samah Ismail Nasef, Chafia Dahou Makhloufi, Elisa Palalane, Hanene Lassoued Ferjani, Ahmed Seri, Doaa Mosad Mosa, Ourida Gacem, Francis Fredrick Furia, Samy Slimani, Christiaan Scott, Djohra Hadef

**Affiliations:** 1 Rheumatology and Rehabilitation, Dept. Faculty of Medicine, Tanta University, Tanta, Egypt; 2 Department of Paediatrics, Aga Khan University Medical College East, P.O Box 30270, Africa NairobiNairobi, 00100 Kenya; 3 Rheumatology Unit, Department of Pediatrics and Child Health, College of Health Sciences, Addis Ababa University, Ethiobia, Ethiopia; 4 University Hospital of Issaad Hassani, Messous, Algeria; 5 Paediatric Rheumatology Unit, Ahmed Gasim Children Hospital, Khartoum, Sudan; 6 Tamale Teaching Hospital, Tamale, Ghana; 7 University of Yaounde, Yaounde, Cameroon; 8 Canterbury Christ Church University, Canterbury, England; 9 Hôpital Riviera-Chablais, Rennaz, Switzerland; 10 Fondation Rhumatismes-Enfants, Lausanne, Switzerland; 11 Department of Rheumatology Kassab Institute, Ur17sp04 Tunis El Manar University Faculty of Medicine of Tunis, Tunis, Tunisia; 12 Haramaya University, Dire Dawa, Ethiopia; 13 Department of Pediatrics, Faculty of Medicine, Mansoura University Children‘S Hospital, Mansoura University, Mansoura, Egypt; 14 Rheumatology Dept. Faculty of Medicine, Benha University, Benha, Egypt; 15 Rheumatology Department, Mongi Slim Hospital, La Marsa, Tunisia; 16 Paediatric Rheumatology Unit, Department of Paediatrics, Lagos State University Teaching Hospital, Ikeja, Lagos, Nigeria; 17 Tripoli Children’S Hospital, University of Tripoli, Tripoli, Libya; 18 Rheumatology Department, Faculty of Medicine, Suez Canal University, Ismailia, Egypt; 19 Department of Rheumatology, Med Lamine Debaghine University Hospital, BD Said Touati, Bab El OuedAlgiers, Algeria; 20 Faculty of Medicine of Algiers, Algiers, Algeria; 21 Hospital Central de Maputo, Maputo, Tunisia; 22 Clinical Immunolgy and Allergy Centre, Royal Care International Hospital / Clinical Immunology and Allergy Department, Soba University Hospital, Khartoum, Sudan; 23 Department of Rheumatology& Rehabilitation, Mansoura University Hospitals, Mansoura, Egypt; 24 Department of Pediatrics, Birtraria Hospital El Biar, University of Algiers 1, Algiers, Algeria; 25 Department of Paediatrics and Child Health, School of Clinical Medicine, Muhimbili University of Health and Allied Sciences, Dar Es Salaam, Tanzania; 26 Atlas Clinic of Rheumatology, Batna, Algeria; 27 Pediatric Rheumatology, University of Ottawa, Ottawa Ontario and University of Cape Twon, Cape Town, South Africa; 28 Faculty of Medicine, Batna 2 University, Batna, Algeria

**Keywords:** JIA, Polyarticular JIA, Guidelines, Pediatric, Africa, PAFLAR

## Abstract

**Background:**

Juvenile idiopathic arthritis (JIA) is the most common rheumatologic disease of childhood. The Existing guidelines for polyarticular JIA are typically based on data from non-African populations and may not fully address the unique challenges faced in African settings. We aimed to produce updated African guidelines for the diagnosis and treatment of children and adolescents with polyarticular juvenile idiopathic arthritis (poly-JIA).

**Methods:**

This study was conducted with the aim of reaching a consensus among African experts on the diagnosis and treatment of poly-JIA using the Delphi technique. The first scientific committee identified a total of 15 key clinical questions according to the PICO (Patient/Population, Intervention, Comparison, Outcome) approach. A systematic review of the evidence-based literature was conducted for this work. The core steering group identified researchers and clinicians with expertise in pediatric rheumatology. A Delphi process was used to reach consensus.

**Results:**

An online questionnaire was sent to the expert panel that participated in the survey (100% response rate). A total of 15 recommendation points were identified, divided into two parts: five recommendations for diagnosis and ten recommendations for management. The percentage of those who agreed with the recommendations (fourth and fifth place) ranged from 80 to 100%. All 15 clinical recommendation statements that the scientific committee had identified had been agreed upon in wording (i.e., 75% of respondents agreed or strongly agreed).

**Conclusions:**

We successfully developed guidelines for children with polyarticular JIA, taking into consideration the African specific nature of limited resources and low income, also on the same time incorporating newly released data and using a treat to target approach.

## Background

Juvenile idiopathic arthritis (JIA) is the most common rheumatologic disease of childhood. JIA is not a single disease but a diagnosis that applies to all forms of arthritis of unknown origin, with an onset before the 16th birthday, lasting more than 6 weeks, and where other causes of arthritis have been excluded [1]. According to the International League Against Rheumatism (ILAR) classification system, there are different clinical categories of JIA, including oligoarthritis, extended oligoarthritis, enthesitis-related arthritis, psoriatic arthritis, polyarthritis (rheumatoid factor (RF) negative), polyarthritis (RF positive) and systemic arthritis [2]. Each form of JIA has a risk of irreversible joint damage and a lower health-related quality of life. The illness can also continue into adulthood, resulting in persistently high morbidity and a reduced quality of life [3]. Polyarticular juvenile idiopathic arthritis (poly-JIA) is a subset of JIA which is characterized by the presence of more than four affected joints during the first six months of disease, poly-JIA affects about 20 to 30% of JIA patients [4, 5].

The main aim of JIA treatment is to achieve remission of the disease with normalization of physical findings and laboratory markers of inflammation, to preserve the physical and psychological integrity of the child, and to prevent any long-term consequences related to the disease or its therapy. Clinical evidence suggests that early diagnosis and treatment of inflammation might improve the prognosis and avert chronic consequences. Treatment recommendations are designed to help healthcare professionals in various manners, such as lowering the likelihood of receiving improper care, promoting the appropriate and efficient use of resources, and encouraging the adoption of a consistent approach to care delivery [6].

The American College of Rheumatology (ACR) published its last recommendations for Non-Systemic Polyarthritis, Sacroiliitis, and Enthesitis in 2019 [7] to provide international guidelines for some JIA subtypes. Africa is a low- and middle-income continent where managing this chronic illness presents major challenges due to scarce resources and inequities. This served as the primary impetus for creating guidelines tailored specifically for African children suffering from JIA. All children JIA have the right to fair access to the best clinical care possible and setting standards of care is one way to realize this right progressively.

The aim of this work is to release updated guidelines for diagnosis and treatment based on evidence for children and adolescents with poly-JIA, considering the African-specific nature of the varied spectrum of resources and low income, also on the same time incorporating newly released data and using a treat-to-target approach.

## Methods

### Design

A multi-step process technique was employed in the development of the consensus guidelines for poly-JIA. Based on the available clinical data and scientific evidence, the study design was developed. The article complied with the meta-analyses and recommended publishing items for systematic review reporting requirements [8].

### Teams for the development of guidelines

Four teams were involved in this work:

1. Core leading team: this team was made up of six pediatric rheumatologists, the project's scope and initial Patient/Population, Intervention, Comparison, and Outcomes (PICO) [9] clinical questions were developed with guidance from the core team, which also supervised and organized the working group. Ultimately, the core team decided which key concerns to include in the guidelines. The Core Team pre-identified outcomes considered crucial for the systematic literature review for each PICO question. The group was also assigned the task of writing the paper and nominating the expert panel.
2. The team responsible for conducting a literature review, headed by an experienced literature review consultant, completed the data abstraction and literature search and assessed the quality of the evidence.
3. The expert panel, consisting of three pediatric rheumatologists, provided assistance in defining the project's scope and in formulating and optimizing the PICO questions.
4. A voting panel made up of twenty pediatric rheumatologists and adult rheumatologists who are experts and interested in pediatric rheumatology, who voted on the recommendations and helped refine the PICO questions and project scope.

### PICO question formulation

“PICO is a specialized framework used by most researchers to formulate a research question, facilitate literature review, and guide to use evidence-based practice, we need a clear idea of the question which would like to answer”.

The project's original objectives, guiding principles, and pertinent PICO question examples were prepared by the core leadership team. The PICO questions were revised via a virtual meeting where topics were developed and deliberated.

- **Populations:** children with polyarticular JIA (either RF positive or negative).
- **Interventions:** measures for diagnosis, assessment, assessment of the treatment target, and different tools of management of poly-JIA.
- **Outcomes**: including disease and functional outcome measures, disease activity measurements, and disease sequelae.

### Review and searching of scientific literature

The evaluation of the literature was carried out with the assistance of a methodology expert, under the supervision of an experienced literature review consultant, and based on the research questions that were determined to concentrate on the diagnosis and management of poly-JIA. Using the PubMed/MEDLINE, EMBASE, and Cochrane databases, a systematic literature search was conducted to obtain appropriate evidence-based background knowledge for deliberations. The experts responsible for the literature review revised the data abstraction, published recommendations, and quality of evidence rating. They also produced a detailed list of recommendations for the diagnosis and management of poly-JIA, based on their own clinical expertise and the available research evidence.

### Target audience

These guidelines have been developed to provide assistance to healthcare professionals who diagnose and manage JIA patients, mainly paediatric rheumatologists, adult rheumatologists with a special interest in paediatric field, and paediatricians with a special interest in paediatric rheumatology.

### Consensus processing

Two Delphi rounds were held to reach a consensus regarding diagnosing and managing poly-JIA. The structured Delphi method guarantees Every participant's perspective equal weight. Online surveys were employed to complete the Delphi process. Each statement was assigned a score between 1 and 5, where 1 meant "complete disagreement," 2 meant "disagreement," 3 meant "neutral," 4 meant "agreement," and 5 meant "complete agreement." Essentially, disagreement, uncertainty, and agreement are represented by the numerals 1–2, 3, and 4–5. The scientific committee assessed the comments which were added to each statement after each voting session.

## Results

For both rounds, the expert panel (*n* = 20 experts in pediatric rheumatology) received the Delphi form. Among the participants, two were from Europe, and eighteen were from all regions of Africa.

The two rounds were done on the key clinical questions to be included in this work. The response rate of the participants was 100%. Consensus was reached on the inclusion of clinical standards on 95% of the items (i.e., ≥ 75% of respondents strongly agreed or agreed). Comments (excluding minor editing suggestions) were more frequent for the availability of diagnostic tools in African countries such as laboratory markers and musculoskeletal ultrasound on diagnosis and monitoring of the disease status. Diversity of opinion was greatest for the item the opportunity for using biologics and biosimilar medicines in Africa due to socioeconomic and regulatory frameworks issues.

At the end of the two-round survey, we had the final recommendations representing the answers of fifteen PICO questions (five PICO questions regarding the diagnosis, and ten PICO questions regarding the management of poly-JIA).

**Fig. 1:**
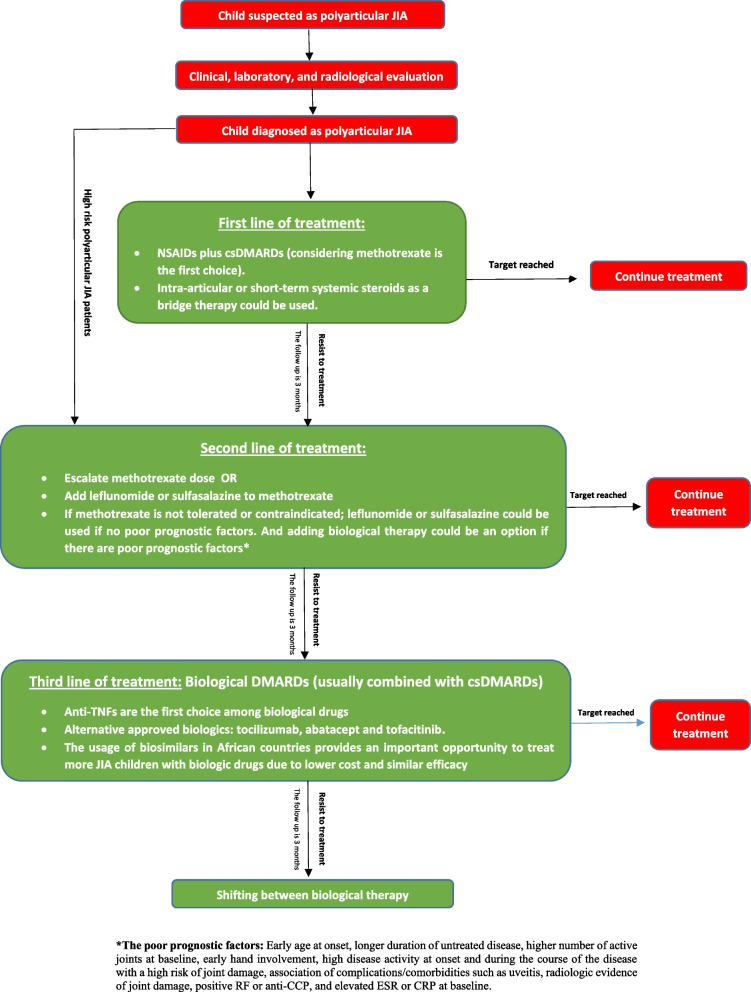
Treatment algorithm for polyarticular JIA

The PICO questions are mentioned in Table 1, the overarching principles are mentioned in Table 2, and Table 3 summarizes the guidelines and levels of evidence, and Fig. 1 demonstrates the treatment algorithm for polyarticular JIA.

**Table 1:** The PICO questions

PICO question Level of agreement on its statement Mean (SD) Percentage of agreement
**Recommendations for diagnosis**
1. When should the practitioner suspect polyarticular JIA and refer the patient to a pediatric rheumatologist?
2. What is the place of imaging studies and which should be performed in the diagnosis of polyarticular JIA?
3. How to diagnose polyarticular JIA?
5. What differential diagnoses should be considered for polyarticular JIA?
6. Are there inflammatory ocular manifestations of polyarticular JIA?
**Recommendations for management**
1. In children with polyarticular JIA, what are treatment targets?
2. In children with polyarticular JIA, what are the non-pharmacological therapies?
3. In children with polyarticular JIA, what is the role of NSAIDs and steroids in management?
4. In children with polyarticular JIA, which therapeutic strategy should be adopted as first-line management (includes consideration of what is available)?
5. In children with polyarticular JIA, which therapeutic strategy should be adopted as second-line management?
6. In children with polyarticular JIA, which therapeutic strategy should be adopted as third-line management?
7. In children with polyarticular JIA, Which Biologics are approved / available?
8. In children with polyarticular JIA, are there high-risk patients who should proceed to 2nd line or 3rd line immediately?
9. In children with polyarticular JIA, how should patients be assessed?
10. In children with polyarticular JIA, when can medication be tapered or withdrawn?

*JIA* Juvenile idiopathic arthritis, *SD* Standard deviation

**Table 2:** Overarching principles

A. Diagnosis of polyarticular JIA should be based on the clinical assessment of an expert rheumatologist who may use some radiological and laboratory studies to facilitate the diagnosis and exclude other differential diagnosis
B. Musculoskeletal ultrasound could be used in diagnosis, monitoring the disease activity, and assessment of response to treatment in polyarticular JIA patients
C. The aim of the treatment is to reach clinical and ultrasound remission or low disease activity using best care methods and should be based on a shared decision between the rheumatologist and the child parents
D. Management of polyarticular JIA may need multiple successive therapies either csDMARDs or bDMARDs, according to the disease activity, response to treatment, safety issues, comorbidities and progression of structural damage

**Table 3:** Summary of the guidelines

Recommendations for diagnosis:	LOE	LOA
**Recommendation 1:** Polyarticular JIA should be suspected, and the child should be referred to a pediatric rheumatologist if child was presented with chronic arthritis affecting 5 or more joints	**IIb**	**90% (High)**
**Recommendation 2:** Plain X-rays, MSUS, and MRI are important radiological investigations which help in the diagnosis of polyarticular JIA	**IIb**	**90% (High)**
**Recommendation 3:** Diagnosis of polyarticular JIA is based on diagnosis of expert pediatric rheumatologist after clinical examination and using laboratory and radiological evaluation guided by the 2001 ILAR Classification Criteria for JIA in children who have arthritis affecting 5 or more joints during the first 6 months of disease	**III b**	**100% (High)**
**Recommendation 4:** Polyarticular JIA should be differentiated from other inflammatory and non-inflammatory arthropathies in children	**IIb**	**100% (High)**
**Recommendation 5:** Uveitis may occur in RF-negative poly arthritis especially in ANA positive children	**I b**	**85% (High)**
**Recommendations for management**		
**Recommendation 1:** The main treatment target is to achieve clinical and/or ultrasound remission, with the alternative target of low disease activity is recommended	**III b**	**85% (High)**
**Recommendation 2:** Non-pharmacologic interventions could be used to optimize supportive care in JIA patients using physical therapy, occupational therapy nutrition, and surgical intervention	**IV C**	**95% (High)**
**Recommendation 3:** NSAIDs could be used as first-line for pain relief and symptom management, and corticosteroids could be used in polyarticular JIA as a bridge therapy, intra-articular injection in resisted joints, or as IV infusion (in severe resisted cases)	**II b**	**85% (High)**
**Recommendation 4:** Starting with csDMARDs (MTX is the first choice) should be considered as first-line in patients with polyarticular JIA	**II b**	**85% (High)**
**Recommendation 5:** The choice of second-line management depends on disease activity, response to initial treatments, individual patient characteristics and prognostic factors. Escalation of MTX dose or using combined csDMARDs should be considered, biological therapy could be used as second line of treatment in presence of poor prognostic factors	**IV b**	**85% (High)**
**Recommendation 6:** Biological DMARDs should be adopted as third-line management after failure of csDMARDs as mono or combined therapy	**II b**	**95% (High)**
**Recommendation 7:** In moderate or severe active polyarticular JIA who do not respond to csDMARDs, biological therapy is recommended in the form of: anti-TNFs, tocilizumab, abatacept and tofacitinib. The usage of biosimilars in African countries provides an important opportunity to treat more JIA children with biologic drugs due to lower cost and similar efficacy	**IV b**	**90% (High)**
**Recommendation 8:** High-risk polyarticular JIA patients who should proceed to 2nd line or 3rd line immediately are those who have poor prognostic factors	**III b**	**95% (High)**
**Recommendation 9:** The child should be assessed clinically, radiographically, and functionally to assess disease activity and response to treatment	**III b**	**95% (High)**
**Recommendation 10:** Stop or withdraw medication depends upon response to the medication, tolerance of the administration regimen, and other patient factors	**IV C**	**85% (High)**

*JIA* juvenile idiopathic arthritis, *MSUS* Musculoskeletal ultrasonography, *MRI* magnetic resonance image, *ILAR* International League of Associations for Rheumatology, *RF* Rheumatoid factor, *ANA* anti-nuclear antibody, *NSAIDs* Non-steroidal anti-inflammatory drugs, *DMARDs* Disease-modifying antirheumatic drugs, *MTX* Methotrexate, *TNF* Tumor necrosis factor, *LOE* Level of evidence, *LOA* Level of agreement

### Recommendations for diagnosis

1. **When should the practitioner suspect polyarticular JIA and refer the patient to a pediatric rheumatologist?** agreement level: Mean+SD: 4.45±0.82, agreement percentage: 90%, agreement level: High

### (Polyarticular JIA should be suspected, and the child should be referred to a pediatric rheumatologist if the child was presented with chronic arthritis affecting 5 or more joints) [10].

2. **What is the place of imaging studies, and which should be performed in the diagnosis of polyarticular JIA? [**11, 12**] **agreement level: Mean+SD: 4.4±0.8, agreement percentage: 90%, agreement level: High

### (Musculoskeletal ultrasound, plain X-ray, and MRI are valuable tools helping in the diagnosis of polyarticular JIA)

- **Musculoskeletal ultrasonography (MSUS)** can be used in the diagnosis of polyarticular** JIA** by detecting synovitis, bone erosion, synovial fluid effusion, and inflammation of the periarticular tissues including the tendons.
- **MSUS also could be used (when available) in monitoring disease activity and response to treatment by evaluating **active synovitis by measuring blood flow signals with power Doppler imaging.
- **Plain X-ray** may be helpful to exclude alternative diagnoses, but radiographs are rarely useful in establishing the diagnosis of JIA.
- **MRI** can show signs of synovitis, including synovial thickening, increased intraarticular fluid, and bone marrow edema, but it's not practically used as a routine in JIA diagnosis, especially with the era of MSUS.

3. **How to diagnose polyarticular JIA? **agreement level: Mean+SD: 4.7±0.47, agreement percentage: 100%, agreement level: High

### (Polyarticular JIA diagnosis is based on the clinical diagnosis of an expert rheumatologist, with the help of some laboratory and radiological tools)

- Diagnosis of polyarticular JIA is based on the diagnosis of an expert pediatric rheumatologist after clinical examination and using laboratory and radiological evaluation guided by the 2001 ILAR Classification Criteria for JIA in children who have arthritis affecting 5 or more joints during the first 6 months of disease.
- Despite all mentioned, there is still no definitive diagnostic test for JIA, and it is a diagnosis of exclusion [13].

4. **What differential diagnoses should be considered for polyarticular JIA?** agreement level: Mean+SD: 4.65±0.49, agreement percentage: 100%, agreement level: High

### (Polyarticular JIA should be differentiated from several autoimmune, infections, and malignant diseases)

- Polyarticular juvenile idiopathic arthritis is a subset of juvenile idiopathic arthritis (JIA) that is defined by the presence of more than four affected joints during the first six months of illness [14].
- It should be differentiated from:
  - ◦ Other subtypes of JIA
  - ◦ Malignant diseases especially leukemia
  - ◦ Post-infectious arthritis and rheumatic fever
  - ◦ Polyarthritis associated with other autoimmune disorders such as pediatric SLE, sarcoidosis, pediatric seronegative spondyloarthropathies as juvenile PSA, and reactive arthritis.

5. **Are there inflammatory ocular manifestations of polyarticular JIA? **agreement level: Mean+SD: 4.45±0.89, agreement percentage: 85%, agreement level: High

### (The incidence of inflammatory ocular disorders increased in ANA-positive children)

- Inflammatory ocular disease is an umbrella term that encompasses uveitis, scleritis, and episcleritis. Among these, uveitis is the commonest.
- Uveitis may occur in RF-negative poly arthritis especially in ANA-positive children, while it is very rare in RF-positive polyarthicular JIA [15].

### Recommendations for management

1. **In children with polyarticular JIA, what are treatment targets? **agreement level: Mean+SD: 4.25±0.0.71, agreement percentage: 85%, agreement level: High

### (The main treatment target is to achieve clinical and/or ultrasound remission, with the alternative target of low disease activity is recommended) [16].

- Clinical assessment using JADAS 27 [17] (inactive disease score ≤1, while low disease activity score from 1.1 to 3.8)
- Ultrasound assessment using EULAR- OMERACT scoring system [18].

2. **In children with polyarticular JIA, what are the non-pharmacological therapies?** agreement level: Mean+SD: 4.65±0.58, agreement percentage: 95%, agreement level: High

### (Non-pharmacologic interventions could be used to optimize supportive care in JIA patients using physical therapy, occupational therapy nutrition, and surgical intervention)

- Physical and occupational therapy are recommended for polyarticular JIA children with a risk of functional limitations.
- Eating a healthy, balanced, nutrient-dense diet, with consideration of specific age-appropriate nutritional requirements is recommended.
- Using specific diet, and herbal supplements isn't recommended to treat JIA.
- Surgical intervention is an option such as synovectomy, ossification surgery, or other corrective surgery e.g. for knee misalignment, and joint replacement for destructed joints [19].

3. **In children with polyarticular JIA, what is the role of NSAIDs and steroids in management? **agreement level: Mean+SD: 4.2±0.89, agreement percentage: 85%, agreement level: High

- NSAIDs could be used as the first line for pain relief and symptom management, especially during initiation for two weeks in case of low activity disease status.
- Steroids may be used either systemic or locally injected inside the refractory joints in polyarticular JIA.
- Intra-articular corticosteroids could be used for refractory joints in polyarticular JIA
- A short course of lowest effective dose of oral corticosteroids could be used as bridging therapy during initiation or escalation of therapy in moderate or severe polyarticular JIA
- Using IV infusion of methylprednisolone for a maximum of 3 days may be an option for resistant active polyarticular JIA [16].

4. **In children with polyarticular JIA, which therapeutic strategy should be adopted as first-line management (includes consideration of what is available)? **agreement level: Mean+SD: 4.2±0.89, agreement percentage: 85%, agreement level: High

### (csDMARDs should be considered as the first-line for management of polyarticular JIA)

- Early initiation of DMARD therapy (methotrexate is conditionally recommended over leflunomide or sulfasalazine). In children with low disease activity, escalation of therapy may be needed for complete disease control.
- Early aggressive therapy in patients with one or more poor prognostic factors should be considered [7].

5. **In children with polyarticular JIA, which therapeutic strategy should be adopted as second-line management? **agreement level: Mean+SD: 4.35±0.74, agreement percentage: 85%, agreement level: High

### (In children with polyarticular JIA, the choice of second-line management (combination of other csDMARDs or using bDMARDs) depends on various factors)

- In children with polyarticular JIA, the choice of second-line management depends on various factors, including disease activity, response to initial treatments, and individual patient characteristics and prognostic factors.
- One common approach for second-line management is to escalate the dose of the already used DMARDs.
- If methotrexate is not tolerated or contraindicated; leflunomide or sulfasalazine could be used if no poor prognostic factors
- leflunomide or sulfasalazine could be added on to methotrexate as a second line of treatment if the target is not achieved with methotrexate monotherapy. (combined MTX and leflunomide is still off-label use in JIA due to scanty of data and conflicting results of studies).
- Biological therapy could be used as second line of treatment in presence of poor prognostic factors [16, 20].

6. **In children with polyarticular JIA, which therapeutic strategy should be adopted as third-line management?** agreement level: Mean+SD: 4.6±0.59, agreement percentage: 95%, agreement level: High

### Biological DMARDs should be adopted as third-line management after failure of conventional synthetic disease-modifying anti-rheumatic drugs (csDMARDs) as mono or combined therapy for polyarticular JIA [7].

7. **In children with polyarticular JIA, Which Biologics are approved / available? **agreement level: Mean+SD: 4.2±0.61, agreement percentage: 90%, agreement level: High

### (Several biological therapies as TNFis, IL6i, and JAKi may be used in resistive polyarticular JIA children)

- In moderate or severe active polyarticular JIA who do not respond to csDMARDs, biological therapy is recommended in the form of: anti-TNFs, tocilizumab, abatacept, and tofacitinib.
- TNF inhibitors that are internationally approved for polyarticular JIA are adalimumab, etanercept, and golimumab in patients at least aged 2 years. Infliximab could be used as an alternative to other TNF inhibitors if they aren't available or contraindicated.
- Tocilizumab could be used in children with moderate or severe active polyarticular JIA who resist treatment with anti-TNFs.
- Tofacitinib could be used for the treatment of polyarticular JIA in patients aged 2 years and older.
- Abatacept could be used in children with moderate or severe active polyarticular JIA when the patient has failed one or more of the other biologics to target a different mechanism.
- Before 2 years of age, the safety & efficacy of biological therapy aren't established.
- Although some biological therapy could be used as monotherapy in the treatment of polyarticular JIA, it's recommended to use biologics in combination with csDMARDs [16, 19, 21].
- Using biosimilars in African countries provides an important opportunity to treat more JIA children with biologic drugs due to lower cost and similar efficacy [22].

8. **In children with polyarticular JIA, are there high-risk patients who should proceed to 2**^nd^** line or 3**^rd^** line immediately? **agreement level: Mean+SD: 4.4±0.59, agreement percentage: 95%, agreement level: High

### (High-risk polyarticular JIA patients who should proceed to 2^nd^ line or 3^rd^ line immediately are those who have poor prognostic factors:)

**Disease activity**:

- High disease activity at onset and during the course of the disease with a high risk of joint damage [23].

**Joint involved [**24**]:**

- hip, cervical spine
- longer duration of untreated disease
- ankle or wrist
- A higher number of active joints at baseline

**Age [**25**]:**

- Early age at onset
- longer duration of untreated disease

**Associated complications/comorbidities:**

- Presence of uveitis [16]

**Imaging [**26**]:**

- Children with radiologic evidence of joint damage
- Radiographic changes of carpal length within the first year of diagnosis

**Blood workup [**27**]:**

- Elevated erythrocyte sedimentation rate at baseline is indicative of high disease activity
- Elevated C-reactive protein at baseline
- RF positive and anticitrullinated peptide antibody positivity has a higher risk for joint erosion.

9. **In children with polyarticular JIA, how should patients be assessed?** agreement level: Mean+SD: 4.4±0.59, agreement percentage: 95%, agreement level: High

### (The child should be assessed clinically, radiographically, and functionally to assess disease activity and response to treatment)

- ◦ Clinical assessment should be done using JADAS (e.g. JADAS 27)
- ◦ Radiographic assessment should be done using MSUS (when available).
- ◦ Assessment of limited joint mobility and functional ability/Health-Related Quality of Life (HRQoL) is recommended.

- Assessment of the patient should be done at baseline, after one month, then every 3 or 6 months according to disease activity and disease status.
- Assessment of treatment efficacy should be done at 3 and 6 months from starting therapy [28].

10. **In children with polyarticular JIA, when can medication be tapered or withdrawn? **agreement level: Mean+SD: 4.15±1.08, agreement percentage: 85%, agreement level: High

### (The decision to taper or withdraw medication for children with polyarticular JIA should be carefully considered and made in consultation with a pediatric rheumatologist)

- Stop or withdraw medication depends upon response to the medication, tolerance of the administration regimen, and other patient factors.
- Withdraw medication might be done when there is persistent inactive disease [for > 6 months] [29].

## Discussion

This manuscript presented updated guidelines about polyarticular JIA, these guidelines focused on the polyarticular type as we in the pediatric African League against Rheumatism (PAFLAR) wanted to release separate guidelines for each subtype of JIA as the diagnostic process, workup, complications, prognosis, and management algorithm differs between each JIA subtype. We call these guidelines African guidelines as we consider the socioeconomic status and drug availability in African countries, but indeed these guidelines could be used globally. In this work we intended to represent all African regions in the expert panel, also, we had two eminent European experts in pediatric rheumatology to revise these guidelines and make these guidelines to be suitable to be applied globally.

In this work, we consider the PICO questions and Delphi technique as PICO question formulation can help in forming a question that focuses on the most important issue for a patient, problem, or population. It helps to identify key terms to use in a search for evidence and select results that directly relate to the situation. Delphi offers participants the convenience of anonymity, controlled feedback, flexibility in selecting the statistical analysis, and the ability to gather participants from a broad spectrum of geographical regions [30, 31].

In these guidelines, we tried to answer five PICO questions regarding polyarticular JIA diagnosis as how to suspect disease, what the most important workup methods are, how to diagnose and differentiate polyarticular JIA from other disorders, and the incidence of ocular manifestations with this JIA category. We gave special consideration to MSUS as an important diagnostic tool that can help in the diagnosis, early detection of even subclinical joint affection, also it is important for disease activity monitoring, and treatment response, and predictive value for disease progression, it's bedside workup test and less expensive than MRI^31^, this was supported by the clinical practice treat-to-target Egyptian guidelines for JIA management [16].

Regarding the management of poly-JIA, this work tried to answer ten questions regarding the treatment target, pharmacological and non-pharmacological management, how to choose the lines and sequence of treatment considering the special circumstances of the African countries, the proper methods for child assessment, and how and when to taper the treatment.

Regarding the treatment target, we considered both clinical and ultrasound target of treatment, this treat to target technique is with acceptance of the Egyptian guidelines [16], while MSUS was not among the targets of treatment in the two ACR guidelines [7, 26]. Many factors were considered concerning when to choose the first, second, and third line of treatment, during the Delphi process in these guidelines as the previous recommendations, the most recent drugs approved for management of poly-JIA, and the special socioeconomic circumstances in Africa and the unavailability of some types of drugs in different African countries, so, in this guidelines we add a statement regarding the importance of the biosimilar biological drugs in African countries.

These guidelines differ from the ACR guidelines in some points as these guidelines concentrate on the polyarticular JIA category only, and, this guideline considers diagnosis and management of poly-JIA, not only the management process. In addition, these guidelines included updated approved therapeutics drugs in poly-JIA.

The diversity and experience of the participants, the high levels of agreement achieved, and the agreement with the most recent JIA treatment recommendations released are among the study's main strengths.

There are some limitations in this work, one of them is that the majority of the recommendations were constrained, and the general quality of the evidence was poor. Also, due to the special consideration of African economics and drug availability, this may affect the application of these guidelines in developed countries outside Africa. One of the main limitations of our work is that we didn't include patient research partners; the selection process of patient research partners should consider communication skills, motivation, and constructive assertiveness in a team setting. Patient research partners receive information and training appropriate to their roles. The contribution of patient research partners to projects should be appropriately recognized, including co-authorship when eligible. That's why members started looking for candidates, but it was difficult in our African countries. Firstly, patients had never participated in a procedure like this in Africa before, and it was unclear what role they might play. The choice was made to solely include patients in the final version's review phase to guarantee that the recommendations were understood. Second, there were major difficulties because of language problems. Finding patients or parents who are fluent in English—a necessary skill for offering an autonomous and impartial view on the recommendations—was challenging in North Africa, where English is the second or third language. This restriction emphasizes the need for improved methods in subsequent projects to handle language obstacles and involve patients sooner in the process. Finally, the lack of patient associations caused yet another difficulty because these organizations could have been of great assistance in encouraging patient participation and guaranteeing that their opinions were fairly heard at every stage. This restriction emphasizes how important it is to encourage the growth of patient associations in Africa, as they may be vital in the development of future guidelines by providing organized assistance and promoting patient involvement. Finally, the lack of patient associations constituted yet another difficulty because these organizations could have been of great assistance in encouraging patient participation and guaranteeing that their opinions were fairly heard at every stage of the procedure.

This limitation underscores the need to foster the development of patient associations in Africa, which could play a crucial role in future guideline development by offering structured support and advocacy for patient participation.

**In conclusion,** in this work, we released updated guidelines for children with polyarticular JIA, taking into consideration the African-specific nature of limited resources and low income, also on the same time incorporating newly released data, and using a treat-to-target approach.

## Data Availability

No datasets were generated or analysed during the current study.

## Abbreviations

ACR: American College of Rheumatology
DMARDs: Disease-modifying antirheumatic drugs
HRQoL: Health-Related Quality of Life
IL6i: IL6 receptor inhibitors
ILAR: International League Against Rheumatism
JADAS: Juvenile Arthritis Disease Activity Score
JAKi: Janus kinase inhibitors
JIA: Juvenile idiopathic arthritis
MSUS: Musculoskeletal ultrasonography
NSAIDs: Non-steroidal anti-inflammatory drugs
PAFLAR: Paediatric Society of the African League Against Rheumatism
PICO: Patient/Population, Intervention, Comparison, Outcome
poly-JIA: Polyarticular juvenile idiopathic arthritis
RF: Rheumatoid factor
TNFi: Tumour necrosis factor inhibitors
